# Peridialytic and intradialytic blood pressure metrics are not valid estimates of 44-h ambulatory blood pressure in patients with intradialytic hypertension

**DOI:** 10.1007/s11255-022-03369-0

**Published:** 2022-09-24

**Authors:** Marieta P. Theodorakopoulou, Maria-Eleni Alexandrou, Fotini Iatridi, Antonios Karpetas, Virginia Geladari, Eva Pella, Sophia Alexiou, Maria Sidiropoulou, Stavroula Ziaka, Aikaterini Papagianni, Pantelis Sarafidis

**Affiliations:** 1 Department of Nephrology, Hippokration Hospital, Aristotle University of Thessaloniki, Thessaloniki, Greece; 2 Therapeutiki Hemodialysis Unit, Thessaloniki, Greece; 3 Department of Radiology, Hippokration Hospital, Thessaloniki, Greece; 4 Department of Nephrology, General Hospital “Korgialeneio-Benakeio”, Athens, Greece

**Keywords:** Hemodialysis, Intradialytic hypertension, Hypertension, Blood pressure measurement, ABPM

## Abstract

**Purpose:**

In contrast to peridialytic blood pressure (BP), intradialytic and home BP measurements are accurate metrics of ambulatory BP load in hemodialysis patients. This study assessed the agreement of peridialytic, intradialytic, and scheduled interdialytic recordings with 44-h BP in a distinct hemodialysis population, patients with intradialytic hypertension (IDH).

**Methods:**

This study included 45 IDH patients with valid 48-h ABPM and 197 without IDH. With 44-h BP used as reference method, we tested the accuracy of the following BP metrics: Pre- and post-dialysis, mean and median intradialytic, mean intradialytic plus pre/post-dialysis, and scheduled interdialytic BP (out-of-dialysis day: mean of 8:00am/8:00 pm readings).

**Results:**

In IDH patients, peridialytic and intradialytic BP metrics showed at best moderate correlations, while averaged interdialytic SBP/DBP exhibited strong correlation (*r* = 0.882/*r* = 0.855) with 44-h SBP/DBP. Bland–Altman plots showed large between-method-difference for peri- and intradialytic-BP, but only + 0.7 mmHg between-method difference and good 95% limits of agreement for averaged interdialytic SBP. The sensitivity/specificity and κ-statistic for diagnosing 44-h SBP ≥ 130 mmHg were low for pre-dialysis (72.5/40.0%, κ-statistic = 0.074) and post-dialysis (90.0/0.0%, κ-statistic = − 0.110), mean intradialytic (85.0/40.0%, κ-statistic = 0.198), median intradialytic (85.0/60.0%, κ-statistic = 0.333), and intradialytic plus pre/post-dialysis SBP (85.0/20.0%, κ-statistic = 0.043). Averaged interdialytic SBP showed high sensitivity/specificity (97.5/80.0%) and strong agreement (κ-statistic = 0.775). In ROC analyses, scheduled interdialytic SBP/DBP had the highest AUC (0.967/0.951), sensitivity (90.0/88.0%), and specificity (100.0/90.0%).

**Conclusion:**

In IDH patients, only averaged scheduled interdialytic but not pre- and post-dialysis, nor intradialytic BP recordings show reasonable agreement with ABPM. Interdialytic BP recordings only could be used for hypertension diagnosis and management in these subjects.

**Supplementary Information:**

The online version contains supplementary material available at 10.1007/s11255-022-03369-0.

## Introduction

Patients with kidney failure undergoing hemodialysis present an extremely high risk of cardiovascular morbidity and mortality [1]. Hypertension is the most common modifiable risk factor in chronic kidney disease (CKD) [2] and, as a result, it is present in approximately 85% of chronic hemodialysis patients [3]. In contrast to pre- and post-dialysis blood pressure (BP) measurements that have either no association or non-linear associations with cardiovascular events and mortality [4–6], elevated BP levels recorded with home or ambulatory BP monitoring (ABPM) are independently associated with increased adverse outcomes in this population [7–10]. Thus, recent consensus documents and the last KDIGO guidelines for BP management in CKD recommend more extended use of ABPM or home BP recordings for BP evaluation in this population [11–13].

The majority of hemodialysis patients exhibit a particular pattern of BP trajectory, i.e. a rapid fall during dialysis session and a progressive increase during the interdialytic interval [14, 15]. However, in approximately 5–15% of these individuals, BP exhibits a paradoxical rise during or immediately after the session, a phenomenon known as “intradialytic hypertension” (IDH) [16]. Previous cohort studies have demonstrated that IDH is associated with even higher risk of cardiovascular and all-cause mortality [17–19]. The mechanistic background of this association is not fully understood; however, a prominent mechanism for the increased cardiovascular risk could be the fact that patients with IDH have also persistently higher elevated BP levels over the whole interdialytic interval compared to individuals with BP fall during hemodialysis [20].

As mentioned above, ABPM is considered the gold-standard method for diagnosis and management of elevated BP in hemodialysis, due to high accuracy, strong associations with outcomes [7–10], and its ability to capture short-term BP variability [15, 21, 22]. Despite its advantages, several factors restrict the wide application of ABPM in these patients; thus, there was a call for studies assessing the agreement of different types of readings with ABPM in this population [12]. In a recent study addressing these important issues, we have observed that averaged intradialytic and scheduled interdialytic BP measurements were valid indexes of 44-h ambulatory BP, in a large cohort of hemodialysis patients, whereas pre- and post-dialysis BP were not [23]. However, whether these metrics display similar agreement with ABPM in the population of patients exhibiting the paradoxical intradialytic BP rise has not been yet investigated. Therefore, the aim of this study, was to assess in parallel the agreement of different indexes of peridialytic, intradialytic and scheduled interdialytic recordings with the 44-h interdialytic BP in patients with and without IDH.

## Materials and methods

### Study participants

This analysis used baseline data from the Northern Greek Hemodialysis Network study, a prospective cohort study aiming to explore patterns of BP and related parameters and their associations with hard outcomes in hemodialysis patients. The inclusion and inclusion criteria for this cohort study are described in detail elsewhere [22, 24]. In brief, inclusion criteria included: (1) age > 18 years and (2) end-stage kidney disease treated with a standard thrice-weekly hemodialysis schedule for > 3 months, (3) provision of informed written consent. Exclusion criteria were: (1) chronic atrial fibrillation or other arrhythmia that interfered with execution of a proper ABPM recording; (2) nonfunctional arteriovenous fistula in the contralateral brachial arm area of the one used for vascular access that could interfere with proper ABPM; (3) modification of dry weight or antihypertensive treatment during 1 month prior to enrollment; (4) myocardial infarction (MI), angina pectoris and stroke during 1 month before study initiation; (5) history of malignant disease or other advanced comorbidity resulting in particularly poor prognosis. All evaluations were performed according to the Declaration of Helsinki (2013 Amendment).

For the purpose of this study we divided the population in patients with and patients without IDH. Those with IDH had: (1) an SBP rise ≥ 10 mm Hg from pre- to post-dialysis and (2) post-dialysis SBP levels of ≥ 150 mmHg at the dialysis session when the 48-h recording was performed [20]. In addition, in a sensitivity sub-analysis (Supplemental Tables 2, 3 and Figs. 5–7), we have examined the validity of these metrics in patients with IDH following a definition of an SBP rise > 0 mmHg from pre- to post-dialysis levels at the dialysis session when the 48-h recording was performed.

### Data acquisition and ambulatory blood pressure monitoring

All patients were evaluated before a mid-week dialysis session. Data for each participant were recorded on specific forms and were transferred to a purpose-built electronic data-collecting sheet. We collected information on demographics, anthropometrics, previous medical history, concomitant medication, and dialysis-related parameters. Venous blood specimens were collected for routine hematologic and biochemical laboratory testing.

ABPM was performed with the Mobil-O-Graph device (IEM, Stolberg, Germany), a validated oscillometric device [25, 26], that was previously shown to provide practically identical values with a widely used ABPM monitor [27]. The device was fitted on the non-fistula arm with a cuff of appropriate size and was programmed to last for a complete 48-h standard intra- and inter-dialytic period. BP was recorded every 20 min from 7:00 a.m. to 10:59 p.m. and every 30 min from 11:00 p.m. to 06:59 a.m. Patients were instructed to follow their usual activities and maintain their usual interdialytic weight gain until the next session. ABPM recordings were considered satisfactory and used for the analysis if the following criteria were met: (1) > 80% of recordings were valid, (2) ≤ 2 non-consecutive day-hours with < 2 valid measurements, and (3) ≤ 1 night-hour without valid recording for each 24-h period [28]. Only patients with complete number of recordings for the intradialytic period were included in this analysis. To minimize the possible effect of manual BP measurements, only measurements recorded at the prespecified time intervals at which the device was set to take measurements were used in this analysis.

### Definitions

We assessed the agreement of the following peri-, intra-, and interdialytic SBP and DBP metrics with ambulatory SBP/DBP recorded during a standard interdialytic interval (i.e. 44-h BP):

1. Pre- and post-dialysis BP, evaluated during the same hemodialysis session with the patient at sitting position at the level of brachial artery in the non-fistula arm.
2. Mean intradialytic BP, defined as the mean of 12 recordings obtained during a standard 4-h dialysis session (i.e. immediately after the start of dialysis and every 20 min thereafter) with the Mobil-O-graph device.
3. Median intradialytic BP, defined as the median of 12 recordings obtained during the 4-h session with the Mobil-O-graph device.
4. Mean intradialytic plus pre/post-dialysis BP, defined as the mean of the 14 recordings consisting of pre-, intra- and post-dialytic BP measurements.
5. Scheduled interdialytic BP, defined as (i) the scheduled Mobil-O-graph reading at 8:00 am of the off-dialysis day; (ii) the scheduled Mobil-O-graph reading at 8:00 pm of the off-dialysis day; (iii) the mean of the two above readings (averaged interdialytic BP). If the exact readings of 8:00 am and 8:00 pm were absent it was allowed to use the immediately previous or next reading; if these were also missing, then the patients were excluded from the analysis.

### Statistical analysis

Continuous variables are expressed as mean ± standard deviation (mean ± SD) or median [interquartile range] according to the normality distribution, based on the Kolmogorov–Smirnov or Shapiro–Wilk tests for normality. Categorical variables are presented as absolute frequencies and percentages (*n*, %). The chi-squared test was used for proportions and Student *t* test or Mann–Whitney *U* test, as appropriate, for continuous data to test for between-group differences.

Within each of the two groups we initially performed paired comparisons with the Student’s paired *t* test or Wilcoxon’s Signed Rank test, where appropriate, to test for possible significant differences between levels of the peri-, intra-, and interdialytic BP indexes and 44-h BP. In addition, we performed simple linear regression analysis and calculated the Pearson coefficients of correlation (*r*) between each of the examined index and 44-h BP to assess the validity of the respective indexes. We also constructed Bland–Altman plots, where the difference between the values of each metric and 44-h BP readings was plotted against their average [29]. In diagnostic accuracy analyses we evaluated the sensitivity, specificity, positive predictive value (PPV), and negative predictive value (NPV) for each of the studied BP metrics at prespecified cut-offs of SBP/DBP ≥ 130/80 mmHg in diagnosing 44-h SBP/DBP ≥ 130/80 mmHg, respectively. Concordance between each different BP metric and 44-h BP at the above thresholds was assessed using κ-statistic [30]. Finally, we performed receiver operating characteristic (ROC) analyses of each of the studied BP metrics examined as a continuous variable for the diagnosis of 44-h SBP/DBP ≥ 130/80 mmHg, respectively. The area under the curve (AUC) for each BP metric was calculated as an aggregate measure of diagnostic performance and is presented with 95% Confidence Intervals (CI) [31]. The best cut-off point for BP was assessed based on the Youden Index calculated as: sensitivity + specificity-1. Statistical significance was assumed at a 5% level. Statistical analysis was performed using Statistical Package for Social Sciences version 22.0 (SPSS Inc, Chicago, IL, USA).

## Results

### Baseline characteristics of study participants

The flowchart of study participants included in the present analysis is depicted in Supplemental Fig. 1. From a total of 286 patients met the inclusion/exclusion criteria, 37 patients had invalid 48-h ABPM and another 7 had invalid recordings of the studied index tests. Thus, a total of 242 patients were included in this analysis. From the latter, 45 had IDH and 197 did not. Table 1 presents baseline demographic, clinical, and laboratory characteristics of the study participants. The two study groups were similar in terms of age (64.69 ± 13.30 vs 62.25 ± 14.42; *p* = 0.301), dialysis vintage, and interdialytic weight gain. Furthermore, no significant differences between the two study groups were observed with regards to the prevalence of major comorbidities. The proportion of patients receiving any antihypertensive medication was similar between the two groups; however, the use of CCBs, central acting drugs and loop diuretics was higher in the IDH group. The use of erythropoietin stimulating agents was not different between the two study groups.

**Table 1:** Demographic, clinical, and laboratory characteristics of the study participants

Parameter	IDH group	Non-IDH group	*P* value
*N*	45	197	
Age (years)	64.69 ± 13.30	62.25 ± 14.42	0.301
Female (*n*, %)	14 (31.1%)	77 (39.1%)	0.319
BMI	26.83 ± 5.84	25.37 ± 3.78	0.591
Dialysis vintage (months)	20.0 [53.0]	28.6 [46.0]	0.373
Diabetes mellitus (*n*, %)	16 (35.6%)	55 (27.9%)	0.310
Hypertension (*n*, %)	44 (97.8%)	176 (89.3%)	0.088
Dyslipidemia (*n*, %)	11 (24.4%)	46 (23.4%)	0.876
Coronary artery disease (*n*, %)	5 (11.1%)	23 (11.7%)	0.915
Peripheral vascular disease (*n*, %)	4 (8.9%)	13 (6.6%)	0.588
Stroke (*n*, %)	5 (11.1%)	14 (7.1%)	0.368
Interdialytic weight gain (kg)	1.68 ± 1.09	1.94 ± 1.00	0.128
UF rate (ml/kg per h)	6.03 ± 3.43	7.23 ± 3.45	**0.039**
Laboratory values			
Hemoglobin (g/dl)	11.26 ± 1.17	11.42 ± 1.35	0.457
Serum urea (mg/dl)	134.02 ± 40.05	140.4033.39	0.268
Serum creatinine (mg/dl)	8.45 ± 2.82	8.41 ± 2.55	0.930
Uric acid (mg/dl)	5.77 ± 1.15	6.53 ± 5.47	0.381
Serum sodium (mg/dl)	137.91 ± 3.19	137.40 ± 3.18	0.336
Serum potassium (mg/dl)	4.84 ± 0.68	4.90 ± 0.65	0.572
Serum calcium (mg/dl)	8.81 ± 0.72	9.02 ± 0.71	0.072
Serum phosphate (mg/dl)	5.29 ± 1.69	5.11 ± 1.34	0.445
Parathormone (ng/dl)	216.0 [197.9]	269.0 [239.0]	0.961
Antihypertensive treatment	41 (91.1%)	162 (82.2%)	0.144
ACEI/ARB (*n*, %)	14 (31.1%)	47 (23.9%)	0.312
Aldosterone blockers (*n*, %)	0 (0%)	3 (1.5%)	0.405
Renin inhibitors (*n*, %)	0 (0%)	1 (0.5%)	0.632
CCBs (*n*, %)	32 (71.1%)	81 (41.1%)	** < 0.001**
Beta-Blockers (*n*, %)	22 (48.9%)	104 (52.8%)	0.741
Centrally active agents (*n*, %)	12 (26.7%)	27 (13.7%)	**0.033**
Loop diuretics (*n*, %)	23 (51.1%)	56 (28.4%)	**0.003**
ESA treatment (*n*, %)	40 (88.9%)	155 (78.7%)	0.145

Bold values denote statistical significance at the *p* < 0.05 level. ACEI angiotensin converting-enzyme inhibitor, *ARB* angiotensin II receptor blocker, *CCB* calcium channel blocker, *ESA* erythropoietin stimulating agents, *ESKD* end-stage kidney disease, *IDH* intradialytic hypertension, *UF* ultrafiltration, *URR* urea reduction rate

### Blood pressure levels

The mean values of pre- and post-dialysis, intradialytic, scheduled interdialytic BP recordings, and the 44-h BP are presented in Table 2. As noted in the table, in patients with IDH, pre-dialysis SBP was non-significantly lower than 44-h SBP (143.4 ± 20.0 vs 146.4 ± 18.9 mmHg; *p* = 0.291), whereas post-dialysis SBP was marginally higher (155.8 ± 24.3 vs 146.4 ± 18.9 mmHg; *p* = 0.053). Mean intradialytic, median intradialytic, intradialytic plus pre/post-dialysis SBP, and averaged scheduled interdialytic SBP levels were non-significantly higher compared with 44-h SBP levels. The results were similar for the relevant DBP metrics (Table 2). In patients without IDH, pre-dialysis BP was significantly higher (144.8 ± 24.0/87.4 ± 14.0 mmHg, *p* < 0.001/*p* < 0.001), whereas post-dialysis SBP was lower (125.1 ± 19.4 mmHg, *p* < 0.001) compared to 44-h SBP/DBP levels. No significant differences between 44-h SBP and mean intradialytic, median intradialytic or intradialytic plus pre/post-dialysis SBP were observed; averaged interdialytic BP was slightly higher than 44-h BP.

**Table 2:** Comparison of peridialytic, intradialytic, and scheduled interdialytic recordings with 44-h ambulatory BP (*p* values refer to comparisons between 44-h SBP/DBP and each one of the other metrics) for patients with and without intradialytic hypertension (IDH), respectively

	BP indexes levels (mmHg)	44 h BP levels (mmHg)	*P* value
IDH group			
SBP			
Pre-dialysis SBP	143.4 ± 20.0	146.4 ± 18.9	0.291
Post-dialysis SBP	155.8 ± 24.3		0.053
Mean intradialytic SBP	149.2 ± 19.0		0.324
Median intradialytic SBP	148.4 ± 19.6		0.464
Intradialytic plus pre/post-dialysis SBP	149.3 ± 18.0		0.307
Interdialytic 8 am SBP	149.7 ± 25.1		0.161
Interdialytic 8 pm SBP	148.1 ± 25.0		0.454
Averaged scheduled interdialytic SBP	148.9 ± 22.2		0.111
DBP			
Pre-dialysis DBP	83.2 ± 11.9	84.8 ± 12.9	0.339
Post-dialysis DBP	89.3 ± 15.8		0.106
Mean intradialytic DBP	87.9 ± 13.8		0.051
Median intradialytic DBP	87.4 ± 13.9		0.075
Intradialytic plus pre/post-dialysis DBP	87.7 ± 13.1		0.065
Interdialytic 8 am DBP	85.8 ± 14.1		0.460
Interdialytic 8 pm DBP	87.5 ± 20.0		0.167
Averaged scheduled interdialytic DBP	86.7 ± 15.3		0.120
Non-IDH group			
SBP			
Pre-dialysis SBP	144.8 ± 24.0	130.6 ± 17.5	**< 0.001**
Post-dialysis SBP	125.1 ± 19.4		**< 0.001**
Mean intradialytic SBP	130.7 ± 17.9		0.862
Median intradialytic SBP	129.1 ± 18.3		0.144
Intradialytic plus pre/post-dialysis SBP	131.3 ± 17.4		0.391
Interdialytic 8 am SBP	131.7 ± 23.8		0.328
Interdialytic 8 pm SBP	133.5 ± 23.2		**0.008**
Averaged scheduled interdialytic SBP	132.6 ± 20.5		**0.008**
DBP			
Pre-dialysis DBP	87.4 ± 14.0	77.7 ± 11.6	**< 0.001**
Post-dialysis DBP	78.6 ± 14.3		0.308
Mean intradialytic DBP	80.7 ± 12.0		**< 0.001**
Median intradialytic DBP	80.4 ± 12.2		**< 0.001**
Intradialytic plus pre/post-dialysis DBP	81.0 ± 11.6		**< 0.001**
Interdialytic 8 am DBP	78.3 ± 16.2		0.498
Interdialytic 8 pm DBP	80.0 ± 16.5		**0.007**
Averaged scheduled interdialytic DBP	79.1 ± 14.0		**0.017**

Bold values denote statistical significance at the *p* < 0.05 level

### Correlation analyses

In correlation analyses for patients with IDH (Supplemental Table 1), pre-dialysis SBP/DBP (*r* = 0.537/*r* = 0.616) showed moderate associations, while post-dialysis BP showed no significant association (*r* = − 0.070/0.211) with 44-h SBP/DBP. Mean intradialytic SBP/DBP (*r* = 0.494/0.689), median intradialytic (0.549/0.736) and intradialytic plus pre/post-dialysis SBP/DBP (*r* = 0.483/*r* = 0.683) showed moderate, while interdialytic BP recordings showed strong correlations with 44-h BP; among these metrics, averaged interdialytic BP exhibited the strongest correlation (*r* = 0.882/*r* = 0.855). In patients without IDH, pre- and post-dialysis BP were moderately correlated with 44-h BP. In contrast, mean intradialytic BP, median intradialytic BP, intradialytic plus pre/post-dialysis BP (*r* = 0.739/*r* = 0.763), and averaged interdialytic BP (*r* = 0.856/*r* = 0.805) were strongly correlated with 44-h BP.

### Bland–Altman analyses

**Fig. 1:**
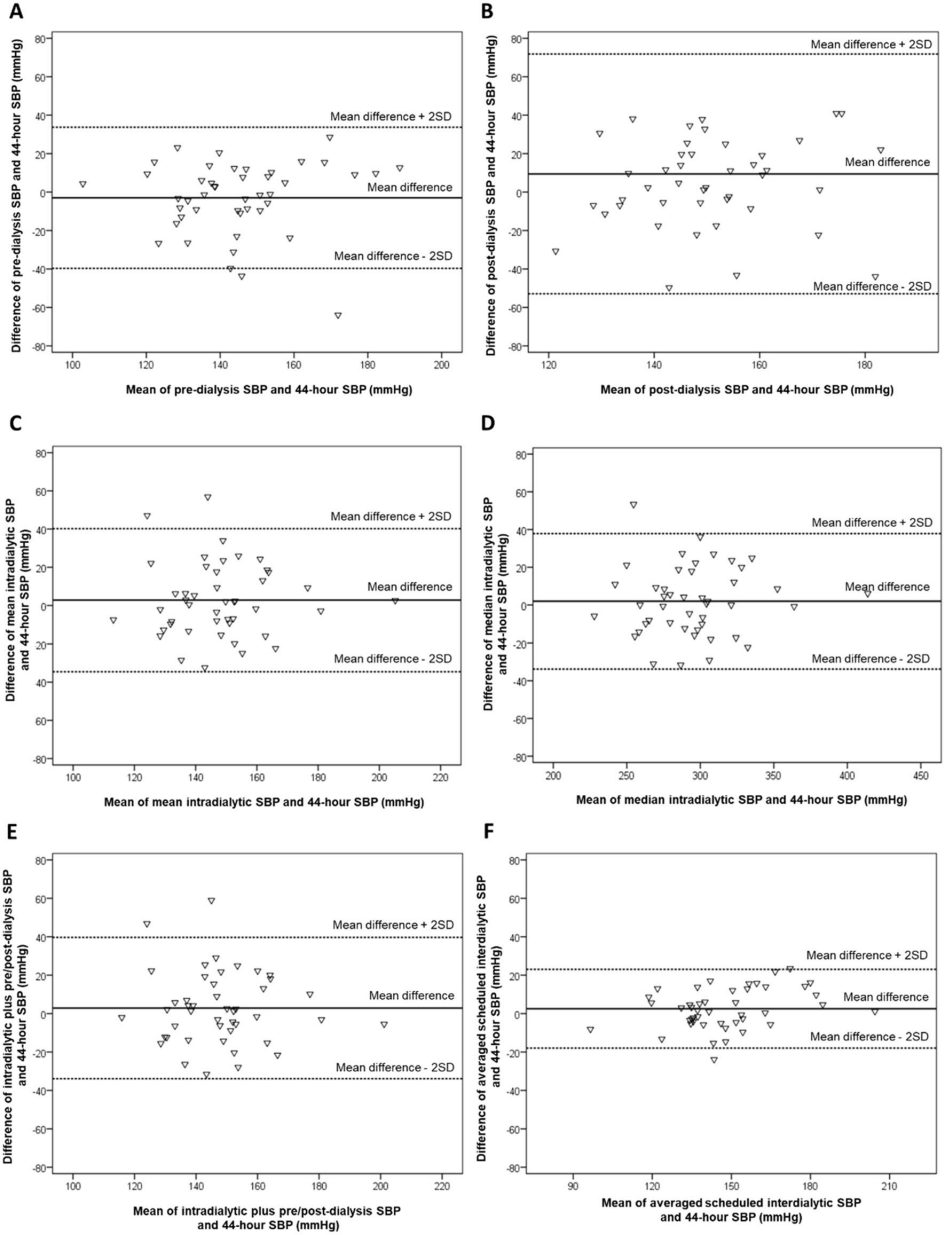
Bland–Altman plots for **A** pre-dialysis SBP, **B** post-dialysis SBP, **C** mean intradialytic SBP, **D** median intradialytic SBP, **E** intradialytic plus pre/post-dialysis SBP, **F** averaged scheduled interdialytic SBP compared with 44-h SBP in patients with intradialytic hypertension

Figure 1 presents the Bland–Altman plots of SBP levels obtained with studied BP metrics against 44-h SBP in patients with IDH. Τhe Bland–Altman plot for pre-dialysis SBP showed a mean between-method difference of −3.0 mmHg with wide 95% limits of agreement (− 39.7 to 33.7 mmHg); the corresponding values were even larger for post-dialysis SBP, with a mean between-method difference of 9.4 mmHg with unacceptably wide 95% limits of agreement (− 52.9 to 71.8 mmHg). With regards to mean and median intradialytic and intradialytic plus pre/post-dialysis SBP, there were relatively small mean between-method differences but again with wide limits of agreement (− 34.6 to 42.2, − 33.84 to 37.86, and − 33.9 to 39.7 mmHg, respectively). Averaged interdialytic SBP showed the smallest between-method difference of + 0.7 mmHg with less wide 95% limits of agreement (− 13.7 to 17.5 mmHg). The relevant Bland–Altman plots for the DBP levels are shown in Supplemental Fig. 2. Again, averaged interdialytic DBP displayed the smallest mean between-method difference and the narrowest limits of agreement among the indexes studied. In patients without IDH, Bland–Altman plots showed large between-method difference and wider 95% limits of agreement for pre- and post-dialysis SBP/DBP compared with mean intradialytic, median intradialytic, intradialytic plus pre/post-dialysis, and averaged scheduled interdialytic SBP/DBP (Supplemental Figs. 3 and 4).

### Diagnostic performance of studied blood pressure metrics

The sensitivity, specificity, PPV, and NPV of each of the studied BP metric at prespecified cut-offs of SBP ≥ 130 and DBP ≥ 80 mmHg in diagnosing 44-h SBP ≥ 130 and DBP ≥ 80 mmHg, along with relevant κ-statistic values, are presented in Table 3. For patients with IDH, the combination of sensitivity and specificity of pre- and post-dialysis, mean intradialytic, median intradialytic and intradialytic pre/post-dialysis SBP/DBP ≥ 130/80 mmHg was not acceptable, whereas all of these metrics showed also the absence of agreement with 44-h BP, as indicated by the low levels of κ-statistic. In contrast, averaged scheduled interdialytic SBP/DBP displayed high sensitivity (97.5/92.0%) and specificity (80.0/75.0%), along with high PPV (97.5/82.1%), NPV (80.0/88.2%) and κ-statistic (0.775/0.680), which were the highest among studied indexes.

**Table 3:** Sensitivity, specificity, positive predictive value, and negative predictive value for each of the studied BP metrics at cut-offs of SBP ≥ 130 and DBP ≥ 80 mmHg in diagnosing 44-h SBP ≥ 130 and DBP ≥ 80 mmHg for patients with and without intradialytic hypertension (IDH), respectively

	Sensitivity (%)	Specificity (%)	Positive predictive value (%)	Negative predictive value (%)	κ-statistic
IDH group					
SBP					
Pre-dialysis	72.5	40.0	90.6	15.4	0.074 (*p* = 0.561)
Post-dialysis	90.0	0.0	87.8	0.0	− 0.110 (*p* = 0.459)
Mean intradialytic	85.0	40.0	91.9	25.0	0.198 (*p* = 0.168)
Median intradialytic	85.0	60.0	94.4	33.3	0.333 (*p* = 0.018)
Intradialytic plus pre/post-dialysis	85.0	20.0	89.5	14.3	0.043 (*p* = 0.771)
Averaged scheduled interdialytic	97.5	80.0	97.5	80.0	0.775 (*p* < 0.001)
DBP					
Pre-dialysis	80.0	70.0	76.9	73.7	0.503 (*p* < 0.001)
Post-dialysis	84.0	40.0	63.6	66.7	0.250 (*p* = 0.070)
Mean intradialytic	88.0	50.0	68.8	76.9	0.394 (*p* = 0.005)
Median intradialytic	88.0	50.0	68.8	76.9	0.394 (*p* = 0.005)
Intradialytic plus pre/post-dialysis	88.0	45.0	66.7	75.0	0.344 (*p* = 0.013)
Averaged scheduled interdialytic	92.0	75.0	82.1	88.2	0.680 (*p* < 0.001)
Non-IDH group					
SBP					
Pre-dialysis	92.1	38.5	61.2	82.2	0.310 (*p* < 0.001)
Post-dialysis	52.5	77.1	70.7	60.7	0.294 (*p* < 0.001)
Mean intradialytic	74.3	78.1	78.1	74.3	0.523 (*p* < 0.001)
Median intradialytic	68.3	84.4	82.1	71.7	0.525 (*p* < 0.001)
Intradialytic plus pre/post-dialysis	73.3	75.0	75.5	72.7	0.482 (*p* < 0.001)
Averaged scheduled interdialytic	84.2	77.1	79.4	82.2	0.613 (*p* < 0.001)
DBP					
Pre-dialysis	86.7	39.5	51.1	80.4	0.239 (*p* < 0.001)
Post-dialysis	77.1	66.7	62.7	80.0	0.425 (*p* < 0.001)
Mean intradialytic	83.1	71.9	68.3	85.4	0.535 (*p* < 0.001)
Median intradialytic	78.3	68.4	64.4	81.3	0.454 (*p* < 0.001)
Intradialytic plus pre/post-dialysis	85.5	72.8	69.6	87.4	0.566 (*p* < 0.001)
Averaged scheduled interdialytic	86.7	76.3	72.7	88.8	0.615 (*p* < 0.001)

κ-statistic indicates concordance between each BP metric and 44-h SBP/DBP at the above thresholds

For patients without IDH, pre- and post-dialysis BP showed low sensitivity/specificity for diagnosing 44-h BP ≥ 130/80 mmHg and poor agreement according to κ-statistic. The corresponding values were higher for mean intradialytic, median intradialytic and intradialytic plus pre/post-dialysis BP. Averaged scheduled interdialytic SBP/DBP displayed a sensitivity of 84.2/86.7% and specificity of 77.1/76.3%, along with the highest PPV, NPV, and κ-statistic (0.613/0.615) among studied indexes.

### ROC analyses

**Fig. 2:**
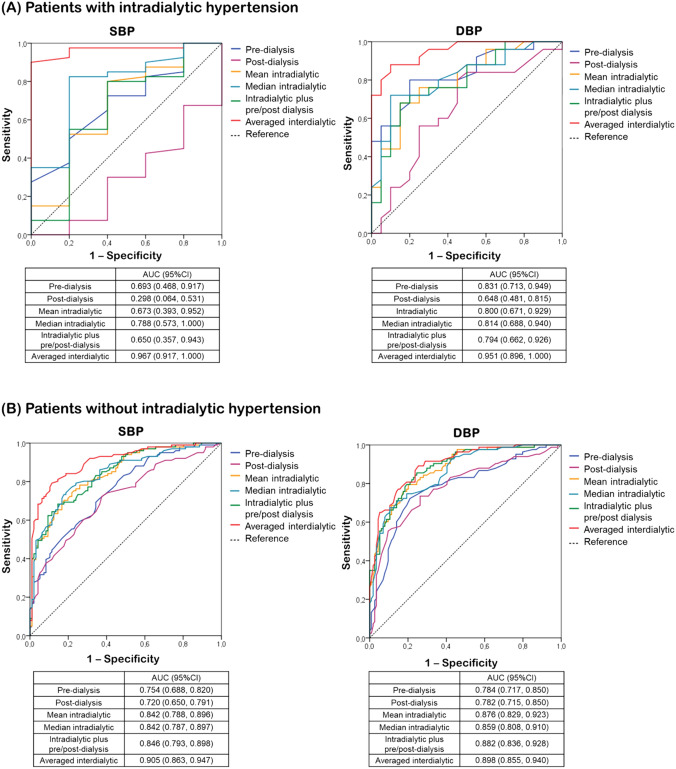
ROC curves of pre-dialysis, post-dialysis, mean intradialytic, median intradialytic plus pre/post-dialysis and averaged scheduled interdialytic BP for the diagnosis of 44-h SBP/DBP ≥ 130/80 mmHg for **A** patients with and **B** patients without intradialytic hypertension (IDH), respectively

Figure 2 presents the ROC curves for studied BP metrics examined as a continuous variable for the diagnosis of 44-h SBP ≥ 130 mmHg and DBP ≥ 80 mmHg, respectively. In patients with IDH, scheduled interdialytic SBP/DBP had the largest AUC (0.967/0.951), along with the highest sensitivity (90.0/88.0%) and specificity (100.0/90.0%) at optimal cut-off values of 133.0/83.5 mmHg (for SBP/DBP, respectively); pre- and post-dialysis SBP/DBP, as well as mean intradialytic SBP/DBP indexes showed worse performance with AUC values at 0.693/0.831, 0.298/0.648, and 0.673/0.800, respectively. For the control group, peridialytic BP metrics displayed lower sensitivity/specificity and AUC values than intradialytic BP metrics and scheduled intradialytic BP at the relevant optimal cut-offs.

### Sensitivity analysis

Supplemental Tables 2, 3 and Supplemental Figs. 5–7 depict the corresponding findings of the diagnostic accuracy analyses for patients with any SBP rise > 0 mm Hg from pre- to post-dialysis levels (*n* = 85). Peridialytic and intradialytic BP metrics showed moderate correlations, while averaged interdialytic SBP/DBP exhibited strong correlation (*r* = 0.867/*r* = 0.831) with 44-h SBP/DBP. Bland–Altman plots showed again large between-method-differences with wide 95% limits of agreement for peri- and intradialytic-BP, but only + 1.68 mmHg between-method difference and good 95% limits of agreement for averaged interdialytic SBP (− 19.61 to 22.97 mmHg). The sensitivity/specificity and κ-statistic for diagnosing 44-h SBP ≥ 130 mmHg were moderate for pre-dialysis (70.0/72.0%, κ-statistic = 0.372) and post-dialysis (86.7/52.0%, κ-statistic = 0.406), mean intradialytic (83.3/72.0%, κ-statistic = 0.535), median intradialytic (78.3/80.0%, κ-statistic = 0.534), and intradialytic plus pre/post-dialysis SBP (80.0/68.0%, κ-statistic = 0.459). Averaged interdialytic SBP showed high sensitivity/specificity (88.3/80.0%) and strong agreement (κ-statistic = 0.668) with 44-h SBP. In ROC analyses, scheduled interdialytic SBP/DBP had again the highest AUC (0.928/0.949) among studied indexes.

## Discussion

This is the first study designed to investigate the accuracy of peri-, intradialytic, and scheduled interdialytic BP recordings in detecting 44-h BP ≥ 130/80 mmHg in patients with IDH. Our main findings were that peri- and intradialytic BP recordings were suboptimal surrogates of 44-h BP, whereas the average of 8 am and 8 pm interdialytic recordings showed strong correlations in simple regression analyses, as well as minimal between-method difference and acceptable 95% limits of agreement in Bland–Altman analyses. Furthermore, scheduled interdialytic BP showed the highest sensitivity/specificity values, the best agreement according to κ-statistic, and the highest values of area under the ROC curve for 44-h ambulatory BP. The above suggest that, in contrast to the typical hemodialysis population, in patients with IDH only scheduled measurements in the out-of-dialysis day are valid surrogate indexes of 44-h BP and could represent alternative methods for diagnosis and treatment of elevated BP.

In the typical hemodialysis population, peridialytic BP recordings are poorly reproducible [11, 32] and have either no association or non-linear associations with cardiovascular outcomes [4–6]. A previous meta-analysis of studies assessing the agreement between peridialytic BP readings with 44-h ABPM indicated that pre-dialysis generally overestimates and post-dialysis underestimates the 44-h BP load [33]. Moreover, in a recent work which is the largest study in the field, it is showed that pre- and post-dialysis BP readings are inaccurate metrics of 44-h BP, and they also have the worst agreement and the lowest sensitivity and specificity for ambulatory BP, among several peri-, intra- and interdialytic BP metric examined [23]. Home BP measurements is long considered another valid option for assessment of BP levels [10, 11, 34, 35]. In hemodialysis subjects, preliminary evidence suggests that home BP readings are more accurate indexes of interdialytic BP [36], and display better associations with adverse outcomes and target-organ damage (i.e. LVH) [8, 9, 37], compared to peridialytic metrics. Moreover, we have previously showed that scheduled awake ambulatory BP readings are also accurate indexes of 44-h BP levels and they could offer an alternative method to identify elevated ambulatory BP in hemodialysis [23]. Given the several factors limiting the widespread use of 48-h ABPM, some previous seminal studies propose that the average of intradialytic and intradialytic with pre- and post-dialysis BP recordings could be another valid alternative. In particular, in a preliminary study in 135 hemodialysis patients in the United States (90% of which were African–American), intradialytic including pre- and post-dialysis BP was a precise, accurate, and reproducible method for diagnosing high ambulatory interdialytic BP [38]. Our aforementioned study expanded the above findings, as we observed in a larger population that both intradialytic BP and intradialytic plus pre- and post-dialysis BP metrics had good performance in diagnosing high ambulatory BP with AUCs in ROC analysis at 0.850 for SBP and around 0.870 for DBP [23]. The findings of the present study for the control group of non-IDH patients are similar with the observations made in the general hemodialysis population with regards to the accuracy of all the metrics discussed above.

Patients with IDH represent a special group of the general dialysis population, as they exhibit a paradoxical rise in BP levels during or immediately after the dialysis session, i.e. at the post-dialysis BP reading [16]. This paradoxical phenomenon is considered an important factor complicating the diagnosis of hypertension on the basis of peridialytic measurements [11, 20]. Furthermore, in the same subjects, 44-h BP load was found to be significantly higher than in controls without IDH, rendering the use of 48-h ABPM as an almost necessary step in the evaluation of BP in this population [12, 20, 39]. In previous studies examining the association of IDH with ABPM, Van Buren et al. showed that post-dialysis BP was a better correlate with 44-h BP than pre-dialysis [39]. In another small recent study, post-dialysis SBP had the least bias with the daytime ABPM in 29 patients with IDH [40]. However, studies assessing the accuracy and agreement of other BP metrics with 48-h ABPM in individuals with IDH were absent.

In the present study, the findings regarding the accuracy of peridialytic BP measurement for detecting elevated ambulatory BP in patients with IDH are similar with the observations made in the general hemodialysis population [23], as both pre- and post-dialysis BP have low sensitivity and specificity for the diagnosis of high 44-h BP. In contrast to the aforementioned preliminary studies [39, 40], we found that post-dialysis BP showed no correlation in simple linear analyses and the absence of agreement with 44-h BP. Furthermore, in contrast to previous observations in the general hemodialysis population [23], intradialytic BP metrics were found to be inaccurate indexes of the 44-h BP load in patients with IDH. Finally, we showed that only scheduled interdialytic recordings had high accuracy and acceptable agreement with 44-h BP levels. It is important to note that our study we did not include typical home BP recordings with validated oscillometric devices [35], but readings obtained at specific timepoints (8 am and 8 pm) of a single non-dialysis day by the ABPM monitor. Thus, our findings refer to readings obtained outside the dialysis unit that are not typical home BP readings. However, the Mobil-o-Graph monitor is also an oscillometric device, and most experts agree that home BP readings and awake ambulatory BP readings offer to similar information regarding the actual BP levels, while home BP and awake ambulatory BP also share the same thresholds to detect high BP [10, 11, 28, 35].

This study has strengths and limitations. It is the first study examining the accuracy of peri-, intra- and scheduled interdialytic recordings for detecting elevated 44-h BP in patients with IDH. Additional strengths include the examination of several candidate BP metrics and the execution of complex analyses including Bland–Altman plots and ROC curves. One major limitation is that we did not use typical home BP readings, as discussed above; thus, although these scheduled interdialytic readings may be very close to appropriate home BP readings at the relevant time points, our findings on this matter should be further confirmed by studies employing typical home BP. In addition, although we used a careful definition of IDH, it referred only to the particular 48-h recording and the requirement of reproducibility of intradialytic BP rise was not covered by this definition; thus some patients included in the IDH group may not had intradialytic BP rise in most sessions, and some patients with the phenomenon present in other sessions may have not been included in the IDH group. Finally, despite the fact that the prevalence of IDH in our cohort is consistent with prevalence rates reported in relevant literature, the absolute number of patients with IDH in our study is relatively small and might not have enough power to explore the diagnostic agreement between different BP metrics; however, as the sensitivity analysis with larger sample size yielded similar results, general impression is that interdialytic BP would be the most accurate BP metric for detecting high 44-h BP even with a larger sample..

In conclusion, this is the first study investigating the accuracy of peri-, intradialytic, and scheduled interdialytic BP recordings in detecting elevated ambulatory BP in patients with IDH. It showed that, in contrast to the general hemodialysis population, in patients with IDH only scheduled interdialytic BP recordings at the off-dialysis day are strongly correlated with 44-h BP, and show acceptable high specificity and sensitivity for detecting elevated 44-h BP. Pre- and post-dialysis, as well as intradialytic BP recordings displayed suboptimal accuracy for diagnosing 44-h BP levels in this population. Future research efforts are needed to further delineate the complex associations of elevated BP with adverse outcomes in this heavily diseased population.

## Supplementary Information

Below is the link to the electronic supplementary material.

Supplementary file1 (DOCX 2475 KB)
