# Supplementary material for: Peridialytic and intradialytic blood pressure metrics are not valid estimates of 44-h ambulatory blood pressure in patients with intradialytic hypertension
Source: Int Urol Nephrol. 2022 Sep 24;55(3):729–40. doi: 10.1007/s11255-022-03369-0 (PMC9958170; doi:10.1007/s11255-022-03369-0)

**SUPPLEMENTAL MATERIAL**

**Title:** Peridialytic and intradialytic blood pressure metrics are not valid estimates of 44-hour ambulatory blood pressure in patients with intradialytic hypertension

**Running head:** BP measurement in patients with IDH

**Authors:** Marieta P. Theodorakopoulou,^1^ Maria-Eleni Alexandrou,^1^ Fotini Iatridi,^1^ Antonios Karpetas,^2^ Virginia Geladari,^1^ Eva Pella,^1^ Sophia Alexiou,^1^ Maria Sidiropoulou,^3^ Stavroula Ziaka,^4^ Aikaterini Papagianni,^1^ Pantelis Sarafidis^1^

**Affiliations:** 1) Department of Nephrology, Hippokration Hospital, Aristotle University of Thessaloniki, Greece; 2) Therapeutiki Hemodialysis Unit, Thessaloniki, Greece; 3) Department of Radiology, Hippokration Hospital, Thessaloniki, Greece; 4) Department of Nephrology, General Hospital "Korgialeneio-Benakeio", Athens, Greece

**Correspondence:** Pantelis Sarafidis, MD, MSc, PhD, Department of Nephrology, Hippokration Hospital, Aristotle University of Thessaloniki, Konstantinoupoleos 49, GR54642, Thessaloniki, Greece. Tel/Fax: +30 2313 312930. E-mail: [psarafidis11@yahoo.gr](mailto:psarafidis11@yahoo.gr)

**Supplemental Table 1**: Correlation coefficients between 44-h BP levels and the relevant levels obtained with peridialytic, intradialytic and scheduled interdialytic readings.

|  | **IDH group (n=45)** | **Non-IDH group (n=197)** |
| --- | --- | --- |
| **SBP** |  |  |
| Pre-dialysis | 0.537 (p<0.001) | 0.615 (p<0.001) |
| Post-dialysis | -0.070 (p=0.649) | 0.554 (p<0.001) |
| Mean intradialytic | 0.494 (p=0.001) | 0.720 (p<0.001) |
| Median intradialytic | 0.549 (p<0.001) | 0.709 (p<0.001) |
| Intradialytic plus pre/post-dialysis | 0.483 (p=0.001) | 0.739 (p<0.001) |
| Interdialytic 8am | 0.786 (p<0.001) | 0.747 (p<0.001) |
| Interdialytic 8pm | 0.778 (p<0.001) | 0.745 (p<0.001) |
| Averaged scheduled interdialytic | 0.882 (p<0.001) | 0.856 (p<0.001) |
| **DBP** |  |  |
| Pre-dialysis | 0.616 (p<0.001) | 0.552 (p<0.001) |
| Post-dialysis | 0.211 (p=0.164) | 0.539 (p<0.001) |
| Mean intradialytic | 0.689 (p<0.001) | 0.754 (p<0.001) |
| Median intradialytic | 0.736 (p<0.001) | 0.732 (p<0.001) |
| Intradialytic plus pre/post-dialysis | 0.683 (p<0.001) | 0.763 (p<0.001) |
| Interdialytic 8am | 0.775 (p<0.001) | 0.692 (p<0.001) |
| Interdialytic 8pm | 0.763 (p<0.001) | 0.694 (p<0.001) |
| Averaged scheduled interdialytic | 0.855 (p<0.001) | 0.805 (p<0.001) |

**Supplemental Table 2**: Correlation coefficients between 44-h BP levels and the relevant levels obtained with peridialytic, intradialytic and scheduled interdialytic readings for patients with any intradialytic SBP rise >0 mmHg (n=85).

|  | **Value** |
| --- | --- |
| **SBP** |  |
| Pre-dialysis | 0.626 (p<0.001) |
| Post-dialysis | 0.583 (p<0.001) |
| Mean intradialytic | 0.704 (p<0.001) |
| Median intradialytic | 0.694 (p<0.001) |
| Intradialytic plus pre/post-dialysis | 0.723 (p<0.001) |
| Interdialytic 8am | 0.775 (p<0.001) |
| Interdialytic 8pm | 0.761 (p<0.001) |
| Averaged scheduled interdialytic | 0.867 (p<0.001) |
| **DBP** |  |
| Pre-dialysis | 0.488 (p<0.001) |
| Post-dialysis | 0.554 (p<0.001) |
| Mean intradialytic | 0.760 (p<0.001) |
| Median intradialytic | 0.746 (p<0.001) |
| Intradialytic plus pre/post-dialysis | 0.764 (p<0.001) |
| Interdialytic 8am | 0.711 (p<0.001) |
| Interdialytic 8pm | 0.731 (p<0.001) |
| Averaged scheduled interdialytic | 0.831 (p<0.001) |

**Supplemental Table 3.** Sensitivity, specificity, positive predictive value, and negative predictive value for each of the studied BP metrics at cut-offs of SBP≥130 and DBP≥80 mmHg in diagnosing 44-h SBP≥130 and DBP≥80 mmHg for patients with any intradialytic SBP rise >0 mmHg (n=85).

|  | Sensitivity (%) | Specificity (%) | Positive prognostic value (%) | Negative prognostic value (%) | κ-statistic |
| --- | --- | --- | --- | --- | --- |
| **SBP** |  | | | | |
| Pre-dialysis | 70.0 | 72.0 | 85.7 | 50.0 | 0.372 (p<0.001) |
| Post-dialysis | 86.7 | 52.0 | 81.3 | 61.9 | 0.406 (p<0.001) |
| Mean intradialytic | 83.3 | 72.0 | 87.7 | 64.3 | 0.535 (p<0.001) |
| Median intradialytic | 78.3 | 80.0 | 90.4 | 60.6 | 0.534 (p<0.001) |
| Intradialytic plus pre/post-dialysis | 80.0 | 68.0 | 85.7 | 58.6 | 0.459 (p<0.001) |
| Averaged scheduled interdialytic | 88.3 | 80.0 | 91.4 | 74.1 | 0.668 (p<0.001) |
| **DBP** |  | | | | |
| Pre-dialysis | 82.5 | 73.3 | 73.3 | 82.5 | 0.554 (p<0.001) |
| Post-dialysis | 87.5 | 57.8 | 64.8 | 83.9 | 0.444 (p<0.001) |
| Mean intradialytic | 85.0 | 66.7 | 69.4 | 83.3 | 0.510 (p<0.001) |
| Median intradialytic | 85.0 | 60.0 | 65.4 | 81.8 | 0.443 (p<0.001) |
| Intradialytic plus pre/post-dialysis | 87.5 | 64.4 | 68.6 | 85.3 | 0.512 (p<0.001) |
| Averaged scheduled interdialytic | 90.0 | 80.0 | 80.0 | 90.0 | 0.695 (p<0.001) |

**Supplemental Figure 1:** Study flow-chart

**
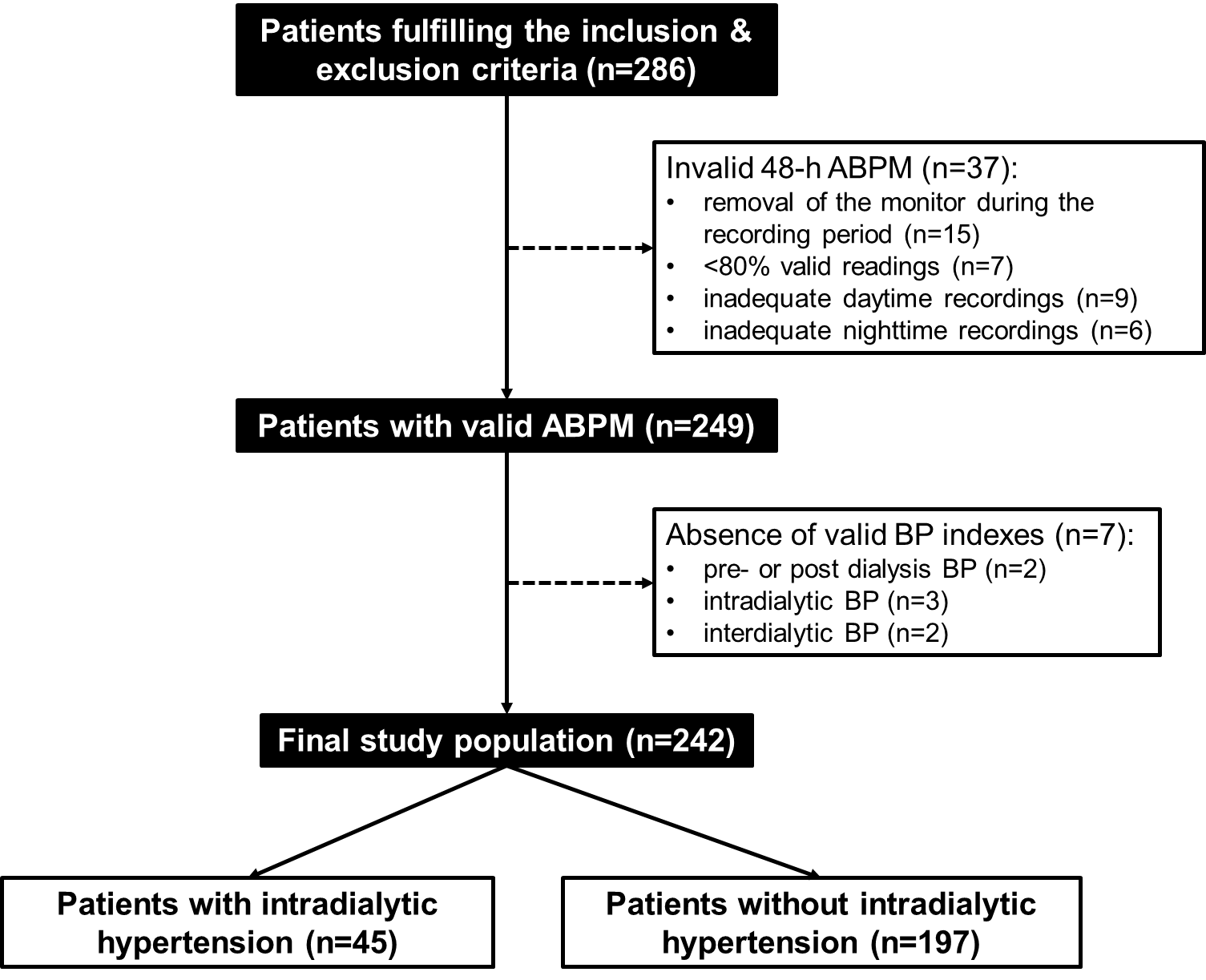
**

**Supplemental Figure 2**: Bland-Altman plots for (A) pre-dialysis diastolic blood pressure (DBP), (B) post-dialysis DBP, (C) mean intradialytic DBP, (D) median intradialytic DBP, (E) intradialytic plus pre/post-dialysis DBP, (F) averaged scheduled interdialytic DBP compared with 44-h DBP for patients with intradialytic hypertension.


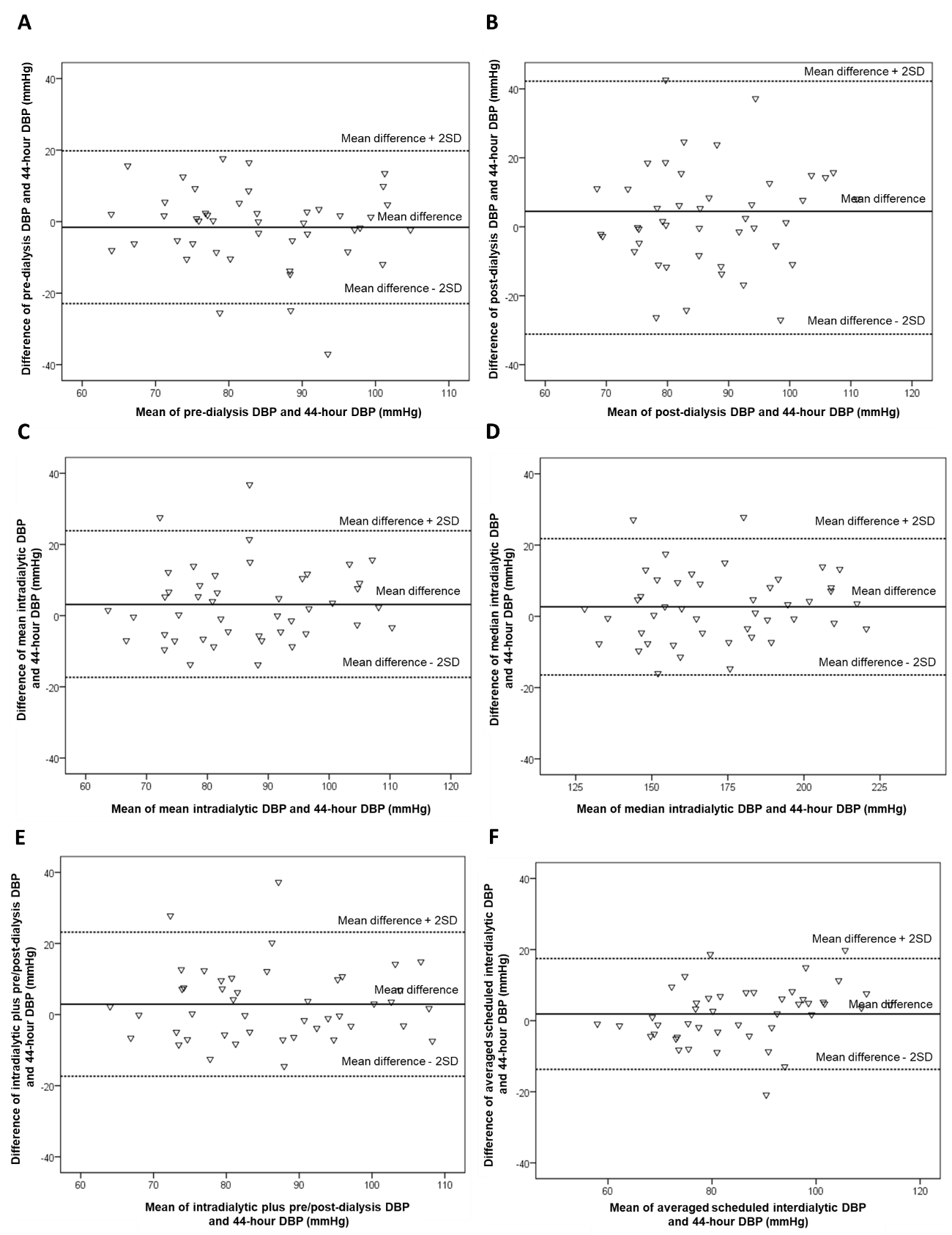


**Supplemental Figure 3**: Bland-Altman plots for (A) pre-dialysis systolic blood pressure (SBP), (B) post-dialysis SBP, (C) mean intradialytic SBP, (D) median intradialytic SBP, (E) intradialytic plus pre/post-dialysis SBP, (F) averaged scheduled interdialytic SBP compared with 44-h SBP for patients without intradialytic hypertension.


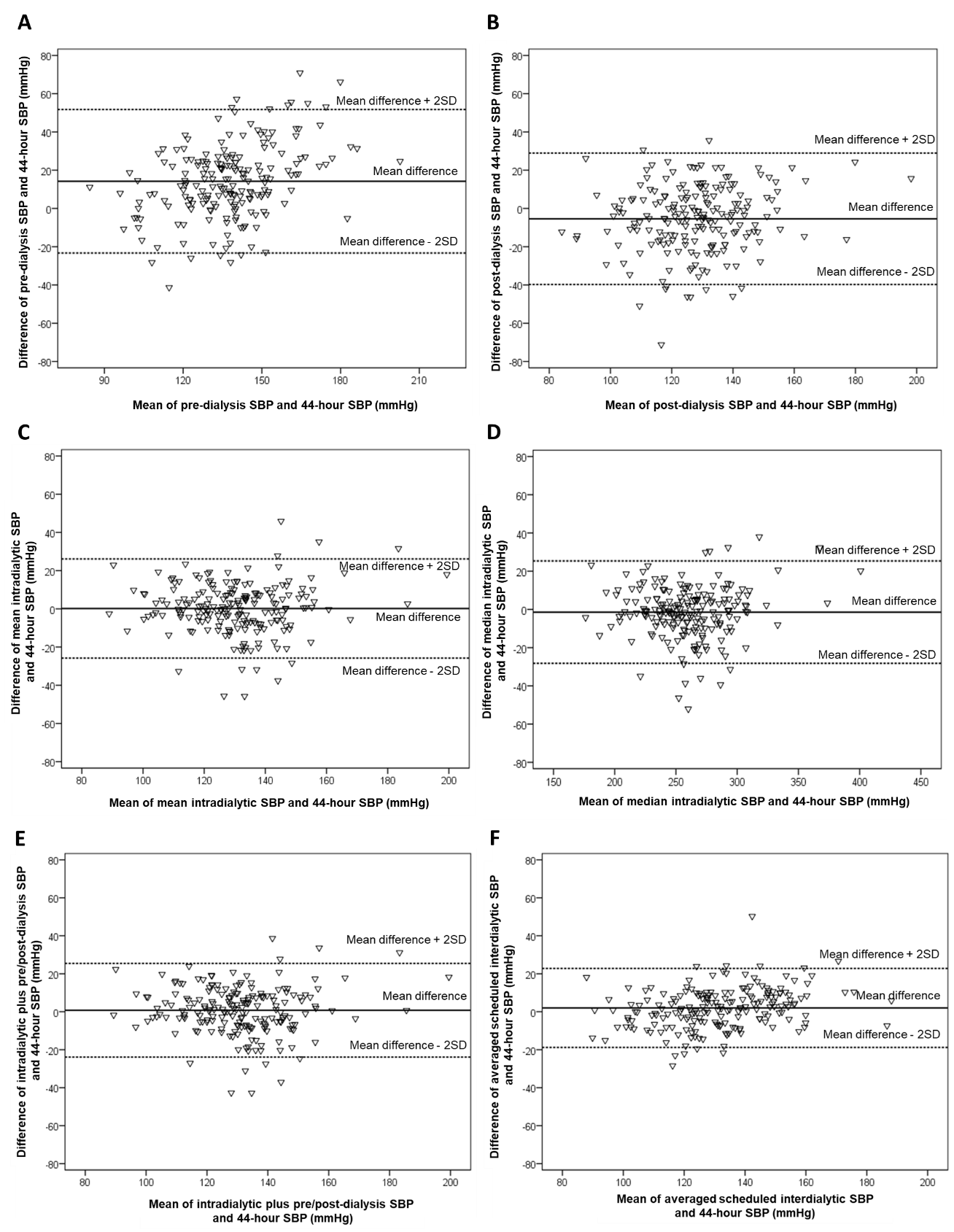


**Supplemental Figure 4**: Bland-Altman plots for (A) pre-dialysis diastolic blood pressure (DBP), (B) post-dialysis DBP, (C) mean intradialytic DBP, (D) median intradialytic DBP, (E) intradialytic plus pre/post-dialysis DBP, (F) averaged scheduled interdialytic DBP compared with 44-h DBP for patients without intradialytic hypertension.


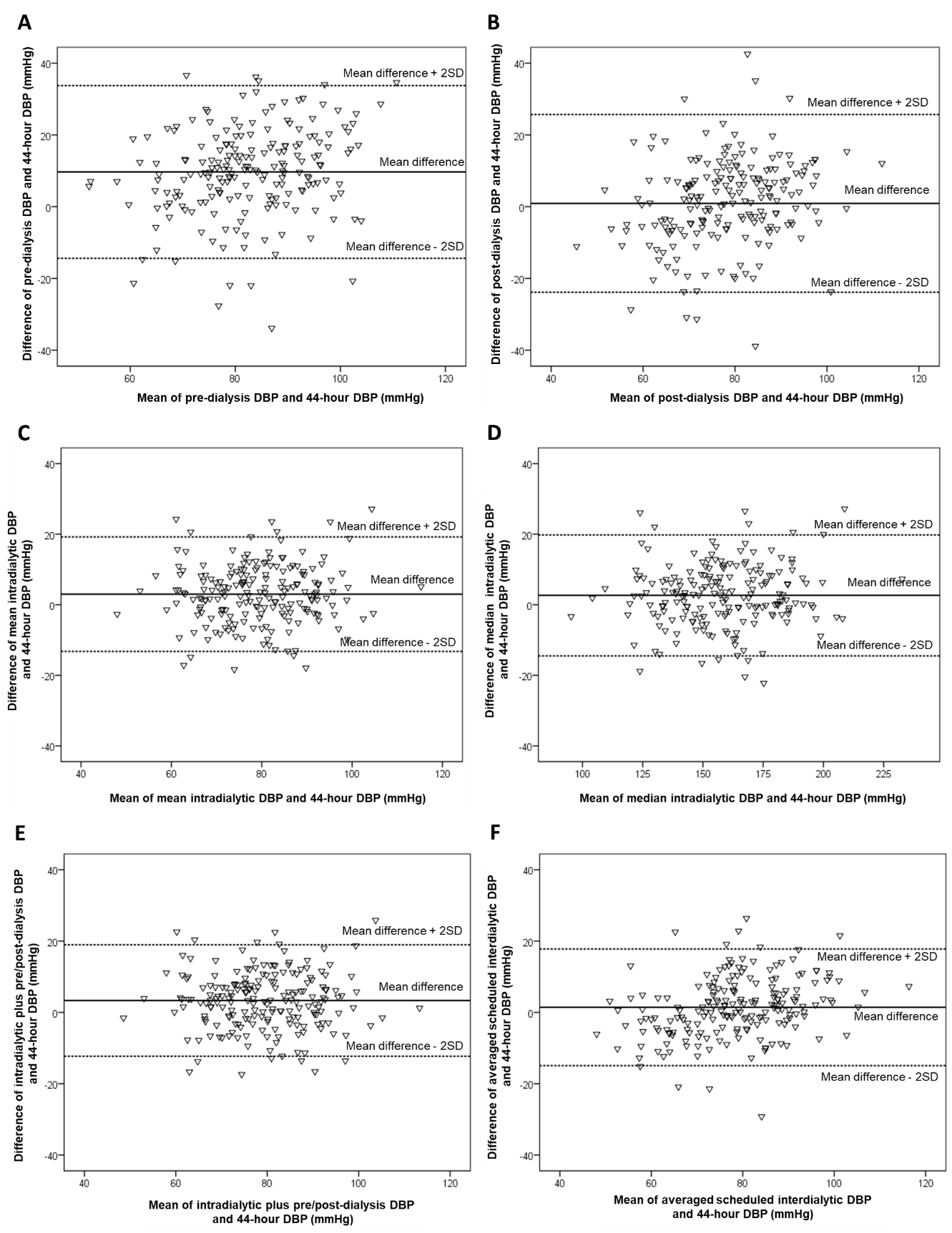


**Supplemental Figure 5**: Bland-Altman plots for (A) pre-dialysis systolic blood pressure (SBP), (B) post-dialysis SBP, (C) mean intradialytic SBP, (D) median intradialytic SBP, (E) intradialytic plus pre/post-dialysis SBP, (F) averaged scheduled interdialytic SBP compared with 44-h SBP for patients with any intradialytic SBP rise >0 mmHg (n=85).

**
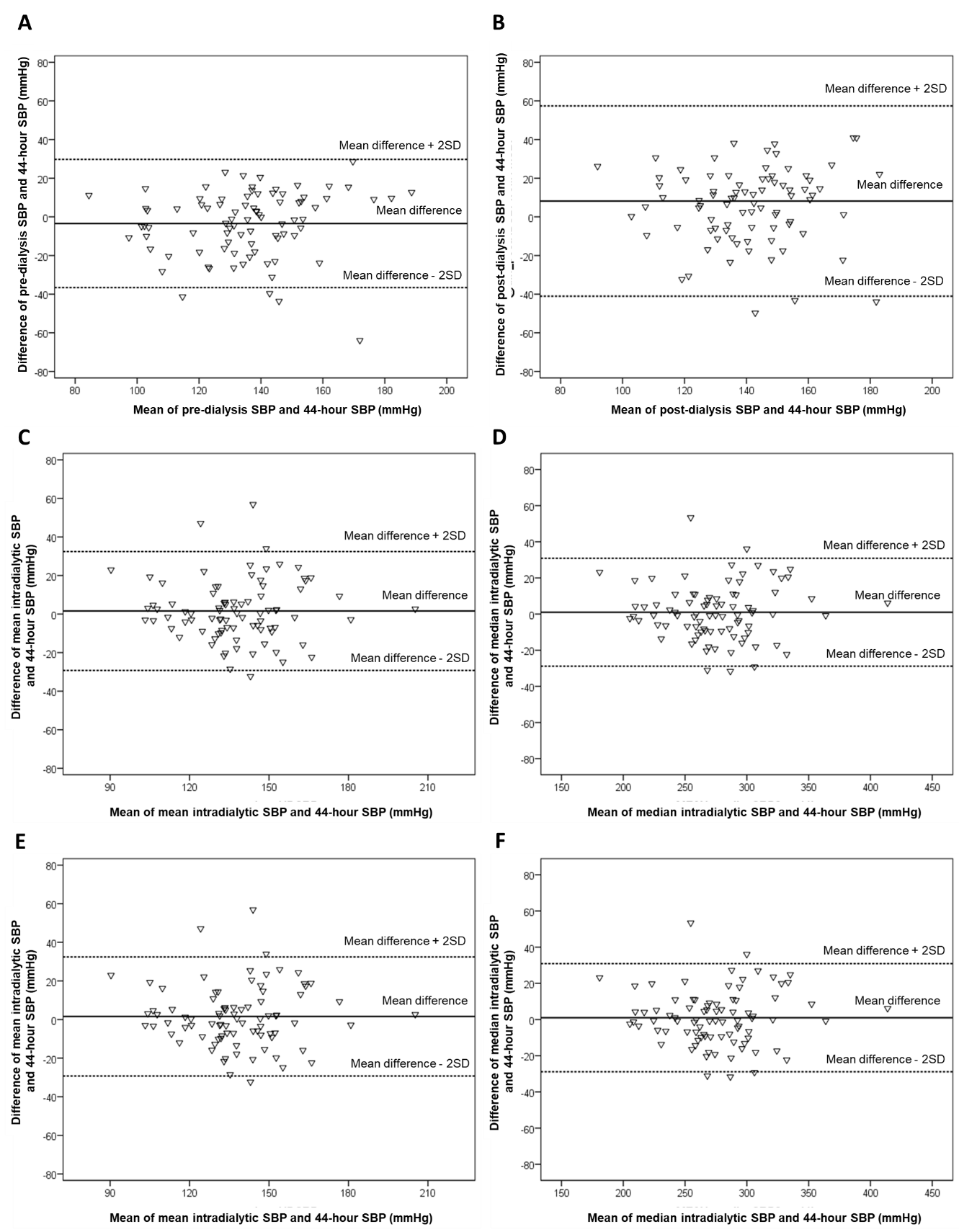
**

**Supplemental Figure 6**: Bland-Altman plots for (A) pre-dialysis diastolic blood pressure (DBP), (B) post-dialysis DBP, (C) mean intradialytic DBP, (D) median intradialytic DBP, (E) intradialytic plus pre/post-dialysis DBP, (F) averaged scheduled interdialytic DBP compared with 44-h DBP for patients with any intradialytic SBP rise >0 mmHg (n=85).

**
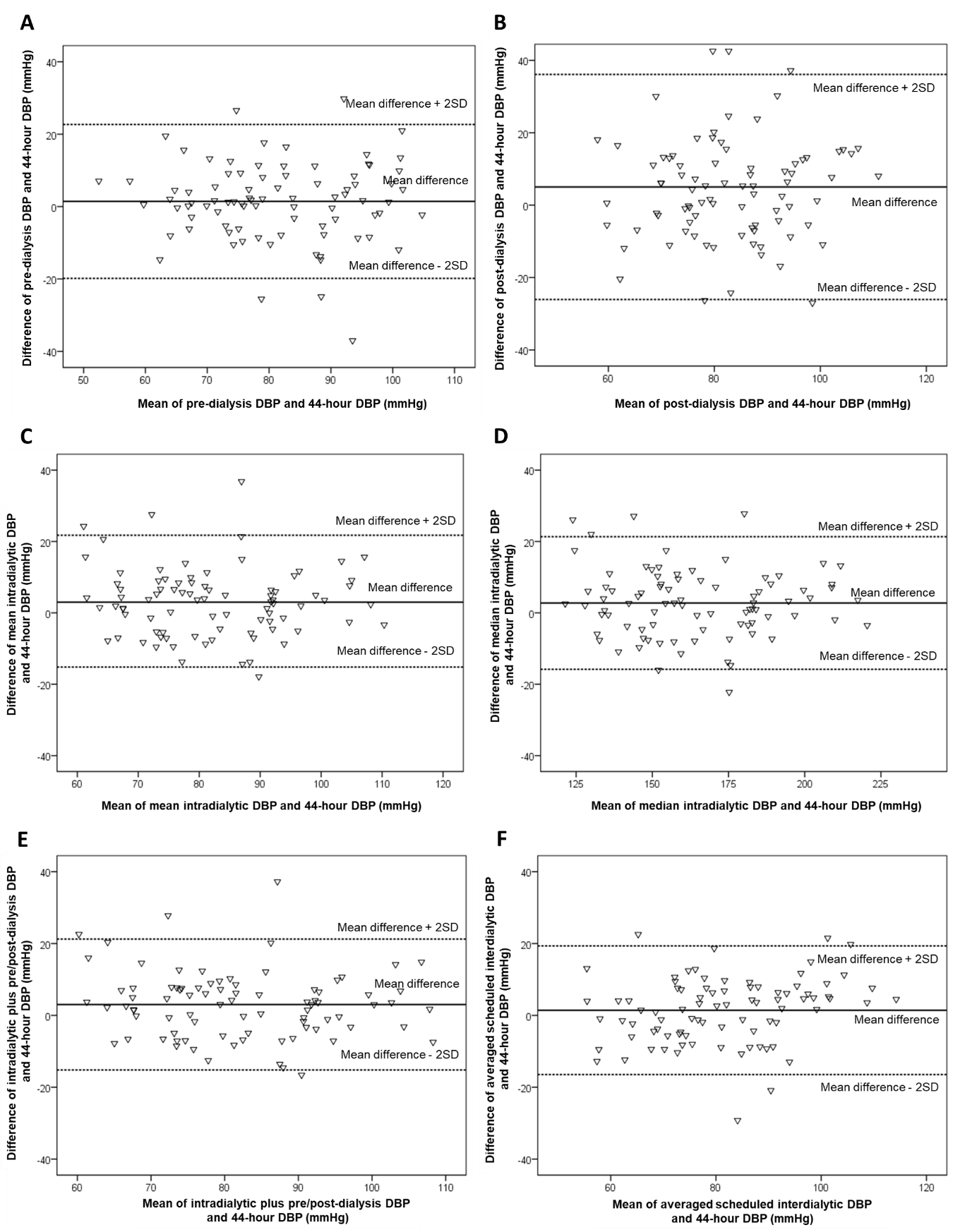
**

**Supplemental Figure 7**: ROC curves of pre-dialysis, post-dialysis, mean intradialytic, median intradialytic, intradialytic plus pre/post-dialysis and averaged scheduled interdialytic BP for the diagnosis of 44-h SBP/DBP≥130/80 mmHg for patients with any intradialytic SBP rise >0 mmHg (n=85).


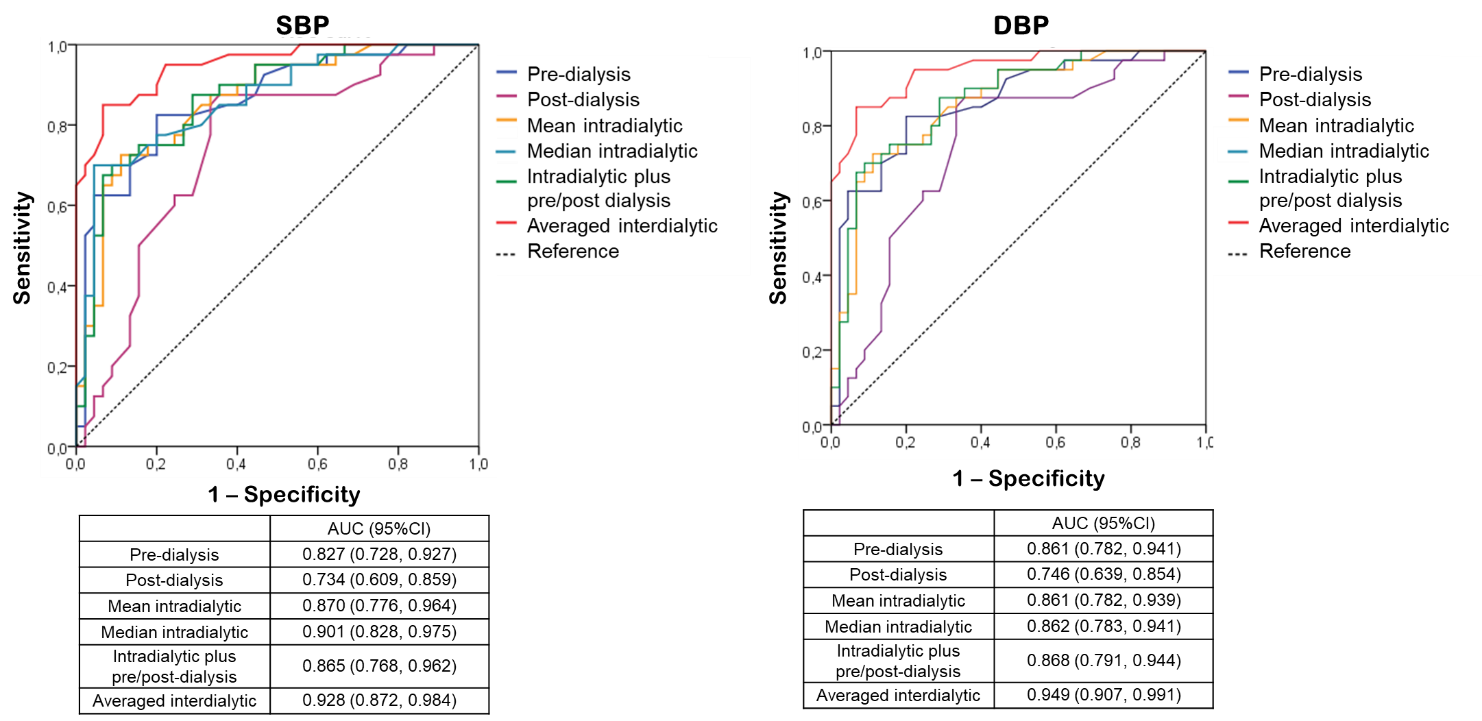

Supplement: Supplementary file 1 — Supplementary file1 (DOCX 2475 KB) [file 11255_2022_3369_MOESM1_ESM.docx]
